# Gas-Phase Pyrolytic Reaction of 4-Aryl-3-buten-2-ols and Allyl Benzyl Ethers: Kinetic and Mechanistic Study 

**DOI:** 10.3390/molecules15010407

**Published:** 2010-01-20

**Authors:** Alya M. Al-Etaibi, Nouria A. Al-Awadi, Maher R. Ibrahim, Yehia A. Ibrahim

**Affiliations:** 1 Natural Science Department, College of Health Science, Public Authority for Applied Education and Training, Kuwait; 2 Chemistry Department, Kuwait University, P.O. Box 5969, Safat 13060, Kuwait

**Keywords:** 4-aryl-3-buten-2-ols, 3-benzyloxy-1-butenes, alkenes, 1,2-dihydronaphthalene, 1,3-butadienes, pyrolysis

## Abstract

Flash vacuum pyrolysis (FVP) of 4-aryl-3-buten-2-ols [ArCH=CH-CH(CH_3_)OH, where Ar is phenyl, *p*-MeO, *p*-Me, *p*-Cl, *p*-NO_2_] gave the corresponding buta-1,3-dien-1-ylbenzene (ArCH=CH-CH=CH_2_, where Ar is Ph, *p*-MeO, *p*-Me, *p*-Cl, *p*-NO_2_) and 7-X-1,2-dihydronaphthalene derivatives (where X is H, MeO); FVP of 1-aryl-3-benzyloxy1-1-butenes and benzyl cinnamyl ethers [ArCH=CHCH(X)OCH_2_Ph, where Ar is phenyl, *p*-MeO, *p*-Me, *p*-Cl, X is H, Me, Ph] gave the corresponding but-2-en-1-ylbenzene derivatives (ArCH_2_CH=CH-X, where X is H, Me, Ph) together with benzaldehyde. The proposed mechanism of these pyrolytic transformations was supported by kinetic and product analysis.

## Introduction

**Scheme 1:**
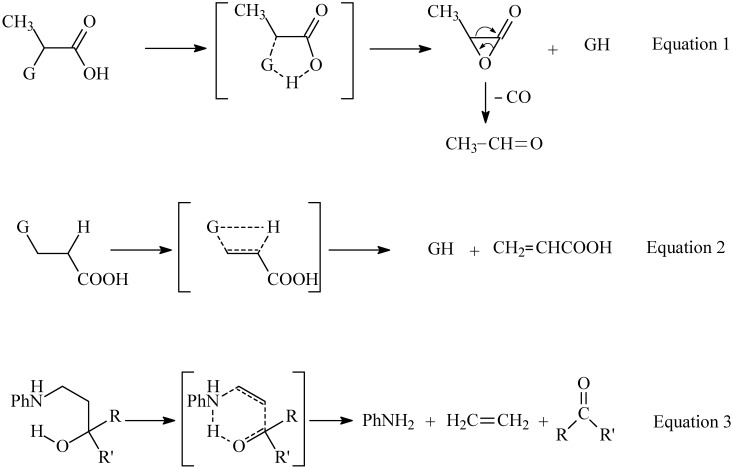
Pyrolysis product and mechanism of gas-phase of α-substituted propanoic acids.

Recently we have shown that gas-phase pyrolysis of α-substituted propionic acids (Scheme 1, where the substituents G = PhO, PhS, PhNH) proceeds by elimination of GH and formation of acetaldehyde and carbon monoxide by the mechanistic pathway presented in Equation 1 [1]. On the other hand, the analogous β-substituted propionic acids undergo retro-Michael elimination to give GH and acrylic acid, as shown in Equation 2 [2]. Moreover, various 3-anilino-1-propanol derivatives have been shown to undergo gas-phase pyrolysis via retro-ene reactions to give anilines, ethylene and carbonyl compounds, as shown in Equation 3 [3,4].

This study reports the gas-phase pyrolytic reactions of 4-aryl-3-buten-2-ols **1a-e** and allyl benzyl ethers **5a-f**. Pyrolysis of **1a-e** is expected to proceed via 1,2-elimination similar to those shown in Equation 2 to produce 1-aryl-1,3-butadienes. On the other hand, pyrolysis of **5a-f** is expected to proceed via a retro-ene mechanism similar to that shown in Equation 3 and which has widely been used in many useful synthetic applications [3,4].

## Results and Discussion

### Synthesis

The required substrates **1a-e, 5a-f** was prepared and fully characterized by NMR and MS, as described in the Experimental section.

### Pyrolysates

**Scheme 2:**
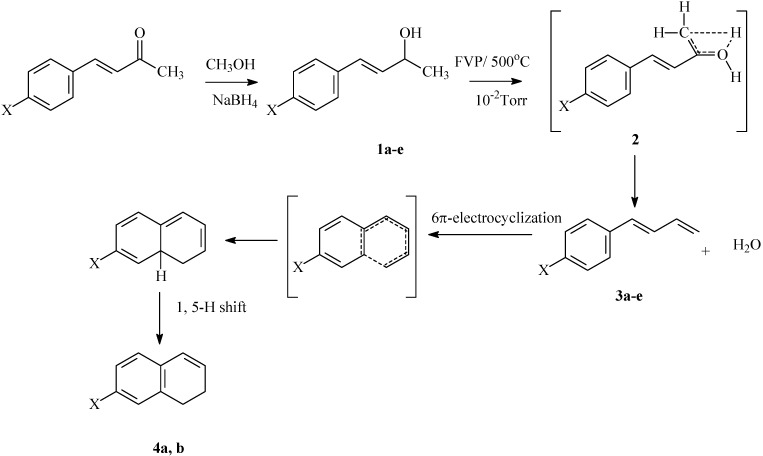
Synthesis and FVP products of 4-aryl-3-buten-2-ols **1a-e**.

Flash vacuum pyrolysis of the 4-aryl-3-buten-2-ols **1a-e** at 500 °C and 10^-2^ Torr afforded the corresponding 1-aryl-1,3-butadienes **3a-e** and 1,2-dihydronphthalenes **4a,b** (Scheme 2). The pyrolysates were qualitatively and quantitatively analyzed by HPLC, the conversion yields were also determined from ^1^H-NMR and the results are recorded in Table 1. The possible mechanistic route for the formation of **3** obtained from the FVP of substrates **1a-e** involves the 4-membered transition state **2**. A similar transition state has been shown to account for the pyrolysis products of β-substituted propionic acids (Equation 2, Scheme 1) [2]. The formation of dihydronaphthalenes **4a,b** involves 6π-electrocyclization of **3a,b** followed by 1,5-H-shift (Scheme 2). The thermal conversion of **3a** to 1,2-dihydronaphthalene **4a** has been previously reported by Badwa [6]. 

**Table 1:** Pyrolysis products of **1a-e** at 500 °C, 10^-2^ Torr and% yield^a^ from FVP.

Cpd	Substrate (X)	Pyrolysis products (% yield)^b^		Recovered substrate
**1a**	H	**3a** (40)	**4a** (50)	**1a** (0)
**b**	OCH_3_	**3b** (38)	**4b** (55)	**2a** (0)
**c**	CH_3_	**3c** (45)^b^	-	**1c** (40)
**d**	Cl	**3d** (68)^b^	-	**1d** (25)
**e**	NO_2_	**3e** (85)^b^	-	**1e** (15)

a) Yields measured by ^1^H-NMR spectroscopy in CDCl_3_ based on the ^1^H doublets at δ 5.23, 5.13, 5.18, 5.23, 5.37 for compounds **3a-e** and 2H multiplets at δ 2.37, 2.30 for **4a,b**; b) This yield refers to conversion yield based on the substrate consumed.

**Scheme 3:**
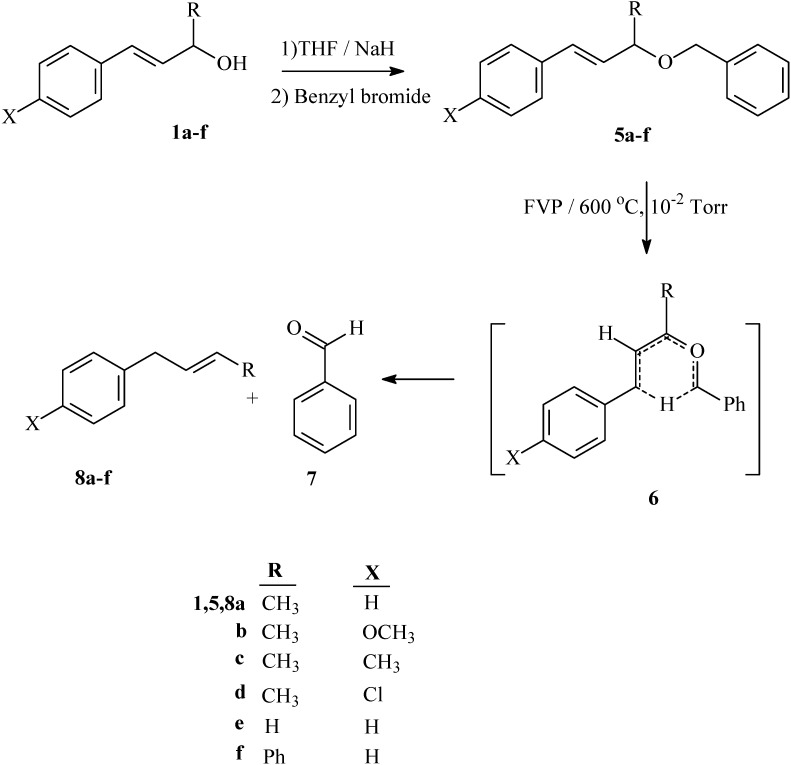
Synthesis and FVP products of allyl benzyl ethers **5a-d**.

Flash vacuum pyrolysis of the allyl benzyl ethers **5a-d** at 600 °C and 10^-2^ Torr afforded but-2-en-1-ylbenzene derivatives **8a-d** in good yield, together with benzaldehyde **7** (Scheme 3). The pyrolysates were qualitatively and quantitatively analyzed by HPLC, yields were also determined from ^1^H-NMR and the results are recorded in Table 2. Similarly, FVP of benzyl cinnamyl ether **5e** and 3-benzyloxy-1,3-diphenylpropene **5f** at 600 °C and 10^-2^ Torr afforded allylbenzene **8e** and 1,3-diphenylpropene **8f**, respectively, together with benzaldehyde (Scheme 3); yields are recorded in Table 2. The possible mechanistic route to the formation of **8a-f** and **7** obtained from the FVP of substrates **5a-f** involves the hetero-retro-ene six-membered transition state **6** (Scheme 3). 

**Table 2:** Pyrolysis products of **5a-f** at 600 °C, 10^-2^ Torr and % yield^a^ from FVP.

Cpd	R	X	Pyrolysis products (% yield)^b^		Recovered substrate
**5a**	CH_3_	H	**8a** (78)	**7** (86)	**5a** (0)
**5b**	CH_3_	OCH_3_	**8b** (91)	**7** (93)	**5b** (0)
**5c**	CH_3_	CH_3_	**8c** (85)	**7** (64)	**5c** (14)
**5d**	CH_3_	Cl	**8d** (68)	**7** (56)	**5d** (20)
**5e**	H	H	**8e** (70)	**7** (69)	**5e** (0)
**5f**	Ph	H	**8f** (60)	**7** (59)	**5f** (0)

a) Yields measured by ^1^H-NMR spectroscopy in CDCl_3_; b) This yield refers to conversion yield based on the substrate consumed.

### Kinetic Analysis

Analysis of the kinetic data reported in Table 3 using the proposed reaction mechanism in Scheme 3 reflects the influence of the substituent group (X) on the reactivity of allyl benzyl ethers **5a-d**. The rate enhancement in going from OCH_3_ > CH_3_ > H > Cl, is systematic and consistent with the electron donating effects of the OCH_3_ on the protophilicity of the π-bond involved in the transition state **6**.

## Experimental

### General

**Figure 1:**
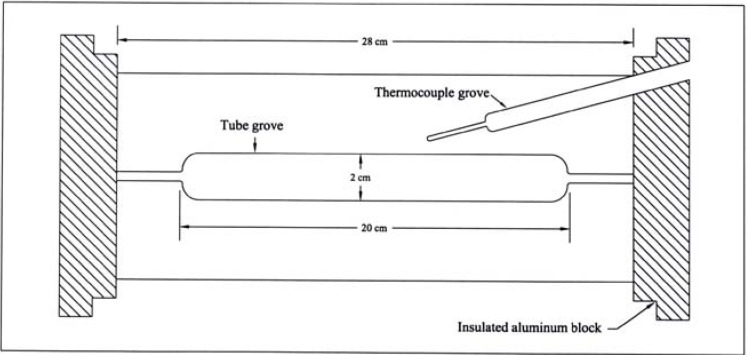
Schematic drawing of the reactor used for kinetic studies by static pyrolysis.

All melting points are uncorrected. IR spectra were recorded in KBr disks on a Perkin Elmer System 2000 FT-IR spectrophotometer. ^1^H- and ^13^C-NMR spectra were recorded on a Bruker DPX 400 MHz super-conducting NMR spectrometer. Mass spectra were measured on VG Autospec-Q (high resolution, high performance, tri-sector GC/MS/MS) and with LCMS using Agilent 1100 series LC/MSD with an API-ES/APCI ionization mode. Microanalyses were performed on a LECO CH NS-932 Elemental Analyzer. The HPLC analysis was performed using a Metrohm HPLC (pump model 7091C and Shimadzu SPD-10 UV/VIS detector), and Supelco LC-8 chromatography column (2.5 cm length × 4.6 mm ID, 5 μm particle size). The reactor used for kinetic and product analysis is a Chemical Data System (CDS) custom-made pyrolyzer consisting of an insulated aluminium-alloy block fitted with a platinum resistance thermocouple connected to a Comark microprocessor thermometer for reactor temperature read-out, accurate to < 0.5 °C (Figure 1). The alloy was chosen for its high thermal conductivity and low temperature gradient, and may be heated for up to 526 °C. The temperature of the reactor is controlled by means of a Eurotherm 093 precision temperature regulator to provide 0.1 °C incremental changes. The reaction tubes were Pyrex: 8 cm long for kinetic runs, and 12 cm for product analysis; with internal and outer diameters 1.5 cm and 1.7 cm, respectively, for both tubes.

### General Procedure of Synthesis of Starting Compounds ***1a-e***

To a cooled solution of the appropriate 4-aryl-3-buten-2-one [7] (10 mmol) in methanol (20 mL) was added portionwise with stirring NaBH_4_ (0.5 g, 15 mmol) [7]. The reaction mixture was then stirred at room temperature for 1 h and the solvent was then removed *in vacuo*. The product was extracted with dichloromethane (DCM, 3 × 50 mL), washed with water and dried over anhydrous sodium sulfate. The solvent was then removed *in vacuo* and the remaining products collected and purified to give **1a-e** [6,7].

*4-Pheny-3-buten-2-ol* (**1a**). Colorless crystals from hexane, mp 40-43 °C, yield 90%; MS: *m/z* = 148 (M^+^, 20%), 130 (100%), 105 (50%); ^1^H-NMR (CDCl_3_): δ 1.40 (d, 3H, *J =* 6.4 Hz), 2.42 (br, 1H, OH), 4.50 (quin, 1H, *J* = 6.4 Hz), 6.29 (dd, 1H, *J* = 15.9, 6.4 Hz), 6.58 (d, 1H, *J =* 15.9 Hz), 7.29 (t, 1H, *J* = 7.2 Hz), 7.36 (t, 2H, *J* = 7.0 Hz), 7.44 (d, 2H, *J* = 7.4 Hz) [6,7].

*4-(4-Methoxphenyl)-3-buten-2-ol* (**1b**). Colorless crystals from pet. ether (60-80), mp 70-73 °C, yield 86%; MS: *m/z* = 178 (M^+^, 30%), 163 (10%), 121 (100%); ^1^H-NMR (CDCl_3_): δ 1.38 (d, 3H, CH_3_ *J* = 6.4 Hz), 1.61 (br, 1H, OH), 3.83 (s, 3H, OCH_3_), 4.48 (quin, 1H, *J* = 6.4 Hz), 6.15 (dd, 1H, *J* = 15.7, 6.4 Hz), 6.53 (d, 1H, *J* = 15.7), 6.87 (d, 2H, *J* = 8.6 Hz), 7.34 (d, 2H, *J* = 8.6 Hz ) [7]. 

*4-(4-Methylphenyl)-3-buten-2-ol* (**1c**). Colorless crystals from pet. ether (60-80), mp 40-42 °C, yield 94%; MS: *m/z* = 162 (M^+^, 10%), 145 (100%), 119 (60%), 105 (60%); ^1^H-NMR (CDCl_3_): δ 1.40 (d, 3H, *J =* 6.4 Hz), 1.58 (br, 1H, OH), 2.36 (s, 3H, CH_3_), 4.50 (quin, 1H, *J* = 6.4 Hz), 6.24 (dd, 1H, *J* = 15.9, 6.4 Hz), 6.56 (d, 1H, *J* = 15.9 Hz), 7.16 (d, 2H, *J* = 7.8 Hz), 7.31 (d, 2H, *J* = 7.8 Hz ); ^13^C-NMR (CDCl_3_): δ 137.6 (C), 133.9 (C), 132.6 (CH), 129.5 (CH), 129.4 (2CH), 126.5 (2CH), 69.1 (C), 23.4 (CH_3_), 21.2 (CH_3_) [7].

*4-(4-Chlorophyl)-2-buten-2-ol* (**1d**). Colorless oil, yield 88%; MS: *m/z* = 182 (M^+^, 90%), 165 (100%), 125 (80%); ^1^H-NMR (CDCl_3_): δ 1.39 (d, 3H, *J* = 6.2 Hz), 1.68 (br, 1H, OH), 4.40 (quin, 1H, *J* = 6.2 Hz), 6.25 (dd, 1H, *J* = 15.9, 6.2 Hz), 6.54 (d, 1H, *J* = 15.9 Hz), 7.29-7.35 (m, 4H) [7].

*4-(4-Nitrophenyl)-3-buten-2-ol* (**1e**). Yellow crystals from hexane, mp 40-42 °C (lit. [9] mp 40 °C), yield 68%; MS: *m/z* = 193 (M^+^, 10%), 175 (100%), 146 (60%); ^1^H-NMR (CDCl_3_): δ 1.42 (d, 3H, *J =* 6.4 Hz), 1.68 (br, 1H, OH), 4.57 (quin,1H, *J* = 6.4 Hz), 6.47 (dd, 1H, *J* = 15.9, 6.4 Hz), 6.68 (d, 1H, *J* = 15.9 Hz), 7.53 (d, 2H, *J* = 8.6 Hz), 8.20 (d, 2H, *J* = 8.6 Hz); ^13^C-NMR (CDCl_3_): δ 23.4 (CH_3_), 68.4 (C), 124.0 (2CH), 126.9 (2CH), 127.0 (CH), 129.4 (C), 138.3 (CH), 143.6 (C). 

### Synthesis of starting compounds ***5a-f**:* General procedure

To a mixture of the appropriate **1a-f** [6,7] (10 mmol) and NaH (60% w/w in mineral oil, 15 mmol) in dry THF (20 mL) was added benzyl bromide (10 mmol). The reaction mixture was heated under reflux for 3 h, the solvent was then removed in vacuo and the residue was extracted with ether (100 mL). The ethereal solution was washed with water (100 mL) and brine (100 mL) and dried over anhydrous Na_2_SO_4_. After removal of the solvent the product was purified by column chromatography using the appropriate solvent to give **5a-f** [9]. 

*(3-Benzyloxybut-1-enyl)benzene* (**5a**). Colorless oil, yield 61%; [R_f_ = 0.84 EtOAc/pet.ether (60-80) 1:7]; MS: *m/z* = 238 (M^+^, 10%), 180 (30%), 146 (10%), 131 (100%); ^1^H-NMR (CDCl_3_): δ 1.60 (d, 3H, *J =* 6.4 Hz), 4.30 (quin,1H, *J* = 6.4 Hz), 4.64 (d, 1H, *J =* 12.0 Hz), 4.82 (d, 1H, *J =* 12.0 Hz), 6.38 (dd, 1H, *J =* 15.9, 6.4 Hz), 6.74 (d, 1H, *J =* 15.9 Hz), 7.38- 7.69 (m, 10H); ^13^C-NMR (CDCl_3_): δ 22.6 (CH_3_), 70.8 (CH_2_), 76.6 (C), 127.3 (2CH), 128.2 (CH), 128.4 (2CH), 128.5 (CH), 129.0 (2CH), 129.4 (2CH), 132.2 (CH), 132.5 (CH), 137.4 (C), 139.6 (C) [9]. 

*1-(3-Benzyloxybut-1-enyl)-4-methoxylbenzene* (**5b**). Yellow oil, yield 68%; [R_f_ = 0.68, EtOAc/pet.ether (60-80) 1:9]; MS: *m/z* = 268 (M^+^, 20%), 252 (30%), 163 (60%). IR: 3063, 2925, 1720, 1453, 1272, 1046, 1070, 751, 699; ^1^H-NMR (CDCl_3_): δ 1.42 (d, 3H, *J =* 6.4 Hz), 3.86 (s, 3H, OCH_3_), 4.13 (quin,1H, *J* = 6.4 Hz), 4.47 (d, 1H, *J =* 12.0 Hz), 4.66 (d, 1H, *J =* 12.0 Hz), 6.07 (dd, 1H, *J =* 15.9, 6.4 Hz), 6.53 (d, 1H, *J =* 15.9 Hz), 6.92 (d, 2H, *J* = 8.4 Hz), 7.30-7.40 (m, 7H); ^13^C-NMR (CDCl_3_): δ 21.9 (CH_3_), 55.2 (CH_2_), 69.9 (OCH_3_), 76.0 (C), 113.9 (2CH), 127.4 (CH), 127.6 (2CH), 127.7 (2CH), 128.3 (2CH), 129.3 (C), 129.4 (CH), 131.0 (CH), 138.8 (C), 159.2 (C); Anal. calc. for C_18_H_20_O_2_ (268.4): C 80.56; H 7.51. Found: C 80.48; H 7.48. 

*1-(3-Benzyloxybut-1-enyl)-4-methylbenzene* (**5c**). Yellow oil, yield 72%; [R_f_ = 0.8 EtOAc/pet.ether (60-80) 1:9]; MS: *m/z* = 252 (M^+^, 10%), 146 (40%), 131 (100%), 105 (50%).IR: 3028, 2925, 1704, 1514, 1454, 1145, 1071, 970, 801, 735, 697 ; ^1^H-NMR (CDCl_3_): δ 1.46 (d, 3H, *J =* 6.4 Hz), 2.43 (s, 3H, CH_3_), 4.17 (quin,1H, *J* = 6.4 Hz), 4.52 (d, 1H, *J =* 12.0 Hz), 4.67 (d, 1H, *J =* 12.0 Hz), 6.19 (dd, 1H, *J =* 15.9, 6.4 *Hz*), 6.58 (d, 1H, *J* = 15.9 Hz), 7.21 (d, 2H, *J* = 8.0 Hz), 7.33- 7.46 (m, 7H); ^13^C-NMR (CDCl_3_): δ 21.2 (CH_3_), 21.9 (CH_3_), 70.0 (CH_2_), 76.0 (C), 126.4 (2CH), 127.4 (CH), 127.8 (2CH), 128.4 (2CH), 129.3 (2CH), 130.7 (CH), 131.4 (CH), 133.9 (C), 137.6 (C), 138.9 (C); Anal. calc. for C_18_H_20_O (252.4): C 85.67; H 7.99. Found: C 85.48; H 7.88. 

*1-(3-Benzyloxybut-1-enyl)-4-chlorobenzene* (**5d**). Yellow oil, yield 64%; [R_f_ = 0.8 ether/pet. ether (60-80) 1:1]; MS: *m/z* = 272 (M^+^, 10%), 214 (25%), 165 (100%), 91 (80%). IR: 3030, 2975, 1703, 1492, 1271, 1096, 1013, 970, 809, 737, 698 ; ^1^H-NMR (CDCl_3_): δ 1.41 (d, 3H, *J =* 6.4 Hz), 4.19 (quin, 1H, *J* = 6.4 Hz), 4.54 (d, 1H, *J* = 12.0 Hz), 4.70 (d, 1H, *J =* 12.0 Hz), 6.24 (dd, 1H, *J =* 15.9, 6.4 *Hz*), 6.59 (d, 1H, *J =* 15.9 *Hz*), 7.39 (m, 5H), 7.46 (m, 4H); ^13^C-NMR (CDCl_3_): δ 21.6 (CH_3_), 70.1 (CH_2_), 75.7 (C), 127.5 (CH), 127.6 (2CH), 127.7 (2CH), 128.3 (2CH), 128.7 (2CH), 130.0 (CH), 132.4 (CH), 133.2 (C), 135.1 (C), 138.7 (C); Anal. calc. for C_17_H_17_O (272.8): C 74.86; H 6.28. Found: C 74.68; H 6.14. 

*3-Benzyl cinnamyl ether* (**5e**). Colorless oil, yield 70%; [R_f_ = 0.8 ether/hexane 5:95]; MS: *m/z* = 225 (M^+^, 20%), 118 (80%); ^1^H-NMR (CDCl_3_): δ 4.30 (d, 2H, *J =* 5.4 Hz), 4.68 (s, 2H), 6.45 (m, 1H), 6.73 (d, 1H, *J =* 15.9 Hz), 7.33-7.52 (m, 10H) [9].

*(3-Benzyloxy-3-phenylprop-1-enyl)benzene* (**5f**). Colorless oil, yield 56%; [R_f_ = 0.76 ether/hexane 1:9]; MS: *m/z* = 300 (M^+^, 20%), 194 (20%), 106 (90%), 104 (80%); IR: 3030, 2925, 1721, 1452, 1270, 1097, 1026, 750,700**;** ^1^H-NMR (CDCl_3_): δ 4.62 (s, 2H ), 5.06 (d, 1H, *J* = 7.2 Hz), 6.38 (dd, 1H, *J* = 16.0, 7.2 Hz), 6.67 (d, 1H, *J* = 16.0 Hz), 7.18 (d, 2H, *J* = 7.2 Hz), 7.23 (m, 2H), 7.25-7.44 (m, 9H), 7.8 (d, 2H, *J* = 7.6 Hz) [11].

### Flash vacuum pyrolysis

The apparatus used was similar to the one which has been described in our recent publication [1]. The sample was volatilized from a tube in a Buchi Kugelrohr oven through a 30 × 2.5 cm horizontal fused quartz tube. This was heated externally by a Carbolite Eurotherm tube furnace MTF-12/38A to a temperature of 500 °C, the temperature being monitored by Pt/Pt-13%Rh thermocouple situated at the center of the furnace. The products were collected in a U-shaped trap cooled in liquid nitrogen. The whole system was maintained at a pressure of 10^-2^ Torr by an Edwards Model E2M5 high capacity rotary oil pump, the pressure being measured by a Pirani gauge situated between the cold trap and pump. Under these condition the contact time in the hot zone was estimated to be =10 ms. The different zones of the product collected in the U-shaped trap were analyzed by ^1^H, ^13^C-NMR, IR and GC-MS. Relative and percent yields were determined from NMR.

### Pyrolysis products

*Buta-1,3-dien-1-ylbenzene* (**3a**). LCMS: *m/z =* 131 (M + 1); ^1^H-NMR (CDCl_3_): δ 5.23 (d, 1H, *J =* 10.0 Hz), 5.39 (d, 1H, *J =* 16.7 Hz), 6.60 (m, 2H), 6.84 (m, 1H), 7.29 (t, 1H, *J =* 7.2 Hz), 7.38 (t, 2H, *J* = 7.4 Hz), 7.41 (d, 2H, *J =* 7.6 Hz) [12].

*1-(Buta-1,3-dien-1-yl)-4-methoxybenzene* (**3b**). LCMS: *m/z =* 161 (M + 1); ^1^H-NMR (CDCl_3_): δ 3.84 (s, 3H, OCH_3_), 5.13 (d, 1H, *J =* 9.0 Hz), 5.30 (d, 1H, *J* = 16.0 Hz), 6.48 (m, 2H), 6.69 (m, 1H), 6.87 (d, 2H, *J =* 8.4 Hz), 7.37 (d, 2H, *J =* 8.4 Hz) [13].

*1-(Buta-1,3-dien-1-yl)-4-methylbenzene* (**3c**). LCMS: *m/z =* 145 (M+1); ^1^H-NMR (CDCl_3_): δ 2.37 (s, 3H, CH_3_), 5.18 (d, 1H, *J* = 10.6 Hz), 5.34 (d, 1H, *J* = 15.4 Hz), 6.56 (m, 2H), 6.78 (m, 1H), 7.19 (d, 2H, *J =* 8.2 Hz), 7.34 (d, 2H, *J* = 8.2 Hz) [14].

*1-(Buta-1,3-dien-1-yl)-4-chlorobenzene* (**3d**). LCMS: *m/z =* 165 (M + 1); ^1^H-NMR (CDCl_3_): δ 5.23 (d, 1H, *J =* 9.6 Hz), 5.43 (d, 1H, *J =* 16.4 Hz), 6.53 (m, 2H), 6.78 (m, 1H), 7.26-7.36 (m, 4H) [15].

*1-(Buta-1,3-dien-1-yl)-4-nitrorobenzene* (**3e**). LCMS: *m/z =* 176 (M + 1); ^1^H-NMR (CDCl_3_): δ 5.37 (d, 1H, *J* = 9.6 Hz), 5.49 (d, 1H, *J =* 15.9 Hz), 6.54 (m, 2H), 6.94 (dd, 1H, *J* = 15.9, 9.6 Hz), 7.53 (d, 2H, *J* = 8.8 Hz), 8.20 (d, 2H, *J* = 8.8 Hz); ^13^C NMR (CDCl_3_): δ 121.0, 124.1, 126.8, 130.4, 134.0, 136.4, 143.6, 146.8 [16].

*1,2-Dihydronaphthalene* (**4a**). LCMS *m/z =* 131 (M + 1); ^1^H-NMR (CDCl_3_): δ 2.37 (m, 2H), 2.86 (t, 2H, *J =* 8.2 Hz), 6.08 (m, 1H), 6.54 (d, 1H, *J* = 8.6 Hz ), 7.07 (d, 1H, *J =* 6.8 Hz), 7.14-7.22 (m, 3H) [17a].

*7-Methoxy-1,2-dihydronaphthalene* (**4b**). LCMS: *m/z =* 161 (M + 1); ^1^H-NMR (CDCl_3_): δ 2.30 (m, 2H), 2.79 (t, 2H, *J =* 8.2 Hz), 3.81 (s, 3H, OCH_3_) 5.91 (m, 1H), 6.55 (d, 1H, *J* = 8.6 Hz ), 6.69 (m, 2H), 6.96 (d, 1H, *J* = 8.6 Hz).

*Benzaldehyde* (**7**). ^1^H-NMR (CDCl_3_): δ 7.58 (t, 2H, *J =* 8.0 Hz), 7.69 (t, 1H, *J* = 7.8 Hz ), 7.94 (d, 2H, *J* = 7.9 Hz), 10.06 (s, 1H) [17b].

*But-2-enylbenzene* (**8a**). LCMS: *m/z =* 133 (M + 1); ^1^H-NMR (CDCl_3_): δ 1.75 (d, 3H, *J* = 6.2 Hz), 3.37 (d, 2H, *J =* 6.2 Hz), 5.61 (m, 2H), 7.22 (t, 2H, *J* = 7.6 Hz), 7.33 (m, 3H) [18].

*1-(But-2-enyl)-4-methoxybenzene* (**8b**). LCMS: *m/z =* 163 (M + 1); ^1^H-NMR (CDCl_3_): δ 1.74 (d, 3H, *J* = 6.2 Hz), 3.31 (d, 2H, *J =* 6.2 Hz), 3.83 (s, 3H, OCH_3_) 5.58 (m, 2H), 6.89 (d, 2H, *J* = 8.4 Hz), 7.15 (d, 2H, *J* = 8.4 Hz) [19].

*1-(But-2-enyl)-4-methylbenzene* (**8c**).LCMS: *m/z =* 147 (M+1); ^1^H-NMR (CDCl_3_): δ 1.75 (d, 3H, *J =* 6.2 Hz), 2.41 (s, 3H, CH_3_), 3.36 (d, 2H, *J* = 6.2 Hz), 5.62 (m, 2H), 7.16 (d, 2H, *J* = 8.2 Hz), 7.43 (d, 2H, *J* = 8.2 Hz) [20].

*1-(But-2-enyl)-4-chlorobenzene* **(8d**. LCMS: *m/z =* 167 (M + 1); ^1^H-NMR (CDCl_3_): δ 1.73 (d, 3H, *J =* 6.2 Hz), 3.31 (d, 2H, *J* = 6.2 Hz), 5.56 (m, 2H), 7.14 (d, 2H, *J* = 8.2 Hz), 7.36 (d, 2H, *J* = 8.2 Hz) [21].

*Allylbenzene* (**8e**. LCMS: *m/z =* 119 (M + 1); ^1^H-NMR (CDCl_3_): δ 3.46 (d, 2H, *J =* 6.2 Hz), 5.17 (m, 2H), 6.05 (m, 1H), 7.27 (d, 2H, *J* = 7.8 Hz*),* 7.39 (t, 2H, *J* = 7.8 Hz*),* 7.45(t, 1H, *J* = 7.8 Hz) [22].

*1,3-Diphenylpropene* (**8f**). LCMS: *m/z =* 195 (M + 1); ^1^H-NMR (CDCl_3_): δ 3.68 (d, 2H, *J =* 6.4 Hz), 6.53 (m, 1H), 6.59 (d, 1H, *J* = 15.8 Hz), 7.28- 7.53 (m, 10H).

### Kinetic runs

Stock solution (7 mL) was prepared by dissolving 5–10 mg of the substrate in acetonitrile to give a concentration of 1 × 10^3^ – 2 × 10^3^ ppm. An internal standard was then added, the amount of which was adjusted to give the desired peak area ratio of substrate to standard (2.5:1). The solvent and standard were selected to be stable under the conditions of pyrolysis, and because they do not interact or react with either substrate or product. The internal standard used in the present study was 1,3-dichlorobenzene and 1,2,4-trichlorobenzene. Each mixture was filtered before use to ensure a homogeneous solution. 

**Figure 2:**
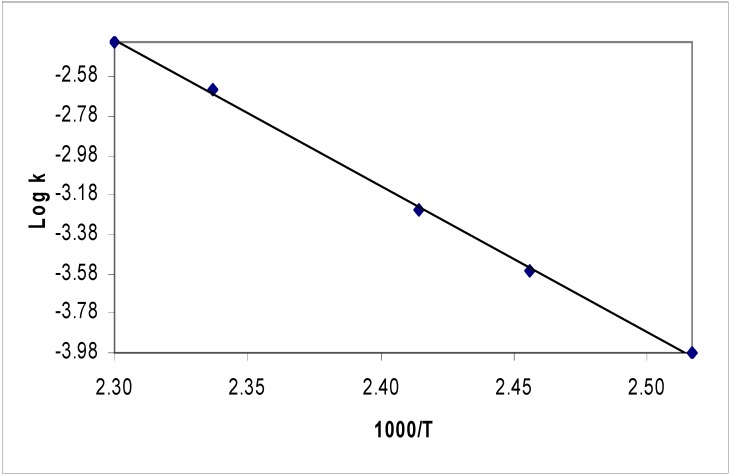
Arrhenius plot for pyrolysis of compounds **5b**.

The ratio of the amount of substrate with respect to the internal standard was calculated from the ratio of the substrate peak area to the peak area of the internal standard. The kinetic rate was obtained by tracing the rate of disappearance of the substrate with respect to the internal standard as follows: An aliquot part (0.2 mL) of each solution containing the substrate and the internal standard was pipetted into the pyrolysis tube, which was then cooled in liquid nitrogen and sealed at reduced pressure (0.20 mbar). The tube was then placed in the pyrolyzer for 6 minutes under non-thermal conditions (ambient temperature). A sample was then analyzed using HPLC with uv/vis detector at wavelength, λ = 256 nm to calculate the standardization value (A_o_). Several HPLC measurements were obtained with accuracy ≥2%. The temperature of the pyrolysis block was then raised until ca 10% pyrolysis of the substrate was deemed to occur over 600 s interval. This process was repeated after each ca 10 °C rise in the reaction temperature until >95% reaction was achieved. The relative ratios of the integration values of the sample and the internal standard (A) at the pyrolysis temperature were then calculated. A minimum of two kinetic runs were carried out at each reaction temperature following each 10 °C rise in the pyrolyzer temperature to ensure reproducible values of (A). Treatment of the kinetic data and calculation of Arrhenius parameters and reaction rate constants have been detailed elsewhere [22]. Figure 2 shows Arrhenius plot for pyrolysis of compounds **5b** as a selected example.

**Table 3:** Rate coefficient (k/s^-1^), Arrhenius parameters of compounds **5a-d.** 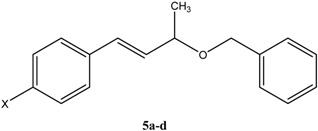

Cpd	X	T /K	10^4^k /s^-1^	log A /s^-1^	Ea / kJ mol^-1^	k_450K_/s^-1^
**5a**	**H**	427.05	1.133	13.48 ± 0.26	258.2 ± 4.88	8.47 × 10^-4^
		440.05	3.477			
		437.15	2.175			
		456.95	14.611			
		466.85	34.889			
**5b**	**OCH_3_**	396.70	1.059	14.62 ± 0.32	141.4 ± 2.37	16.30 × 10^-3^
		406.55	2.764			
		413.55	5.604			
		427.25	23.126			
		434.05	39.985			
**5c**	**CH_3_**	412.75	1.617	20.25 ± 0.57	190.1 ± 4.63	15.39 × 10^-3^
		417.50	2.939			
		422.45	5.222			
		427.45	10.91			
		432.45	19.81			
**5d**	**Cl**	467.15	1.583	7.21 ± 0.34	138.1 ± 6.49	6.60 × 10^-5^
		477.35	3.162			
		487.05	4.737			
		497.05	9.068			
		506.85	12.128			
		516.80	18.945			
		526.45	21.774			

## Conclusions

This work describes pyrolytic synthesis of 1-aryl-1,3-butadienes and 3-arylalkenes. The proposed mechanism is supported by kinetic studies. This represents a general method for the synthesis of such important olefinic derivatives.
